# Objective and Self-Rated Sedentary Time and Indicators of Metabolic Health in Dutch and Hungarian 10–12 Year Olds: The ENERGY-Project

**DOI:** 10.1371/journal.pone.0036657

**Published:** 2012-05-07

**Authors:** Mai J. M. Chinapaw, Mine Yıldırım, Teatske M. Altenburg, Amika S. Singh, Éva Kovács, Dénes Molnár, Johannes Brug

**Affiliations:** 1 Department of Public and Occupational Health and the EMGO Institute for Health and Care Research, VU University Medical Center, Amsterdam, The Netherlands; 2 EMGO Institute for Health and Care Research, VU University Medical Center, Amsterdam, The Netherlands; 3 Department of Paediatrics, University of Pécs, Pécs, Hungary; 4 Department of Epidemiology and Biostatistics, EMGO Institute for Health and Care Research, VU University Medical Center, Amsterdam, The Netherlands; University of Montreal, Canada

## Abstract

**Background:**

The association between objectively assessed sedentary time and metabolic risk factors in childhood have rarely been studied. Therefore, we examined the independent relationship between objectively assessed and self-rated sedentary time and indicators of metabolic health in Dutch and Hungarian 10–12 year olds.

**Methodology/Principal Findings:**

We performed a cross-sectional survey in primary schools. Participants were Dutch and Hungarian girls (n = 73, aged 12.2±0.6 years, 18% overweight/obese) and boys (n = 69, aged 12.2±0.7 years, 38% overweight/obese). Sedentary time and physical activity were assessed by the Actigraph accelerometer. TV and PC time were assessed by self-report. Adiposity indicators included body weight, height, and waist circumference (WC). Fasting plasma glucose, C-peptide, total cholesterol, low density lipoprotein cholesterol, high density lipoprotein cholesterol, and triglycerides were determined in capillary blood and summed into a metabolic risk score. Linear regression analyses were adjusted for physical activity, number of sedentary bouts and WC. Children spent on average 7.6 hours of their daily waking time in sedentary behavior and self-reported 116±64 min/day watching TV and 85±57 min/day using the computer. Comparing the 1^st^ and 4^th^ quartile of objectively assessed sedentary time, C-Peptide levels, WC and BMI were significantly higher in the most sedentary quartile, while the difference in metabolic risk score was borderline significant (p = 0.09). Comparing the 1^st^ and 4^th^ quartile of TV time, BMI was significantly higher in the most sedentary quartile, while the difference in WC score was borderline significant (p = 0.06). In the adjusted linear regression analysis we found no significant association of sedentary time with metabolic risk.

**Conclusions/Significance:**

Although BMI and WC were higher in the most sedentary versus the least sedentary children; we found no further evidence that more sedentary children were at increased metabolic risk.

## Introduction

Sedentary behavior and physical activity are two distinct classes of behavior and evidence is emerging that both have independent effects on weight and metabolic function [1], [2]. Children spend more time with media (TV, videogames and Internet) than any other activity other than sleeping [3], and such media-related activities are in general sedentary. The most direct effect of sitting idle is that the work performed by the large skeletal muscles in the legs, back, and trunk required for upright movement comes to a halt. Over the course of one day, sedentary behavior may induce negative effects on relatively fast-acting cellular processes in skeletal muscles or other tissues regulating risk factors like plasma triglycerides and HDL cholesterol [4]–[6]. Sitting for prolonged periods is also associated with lower energy expenditure increasing the propensity to become overweight [7]. Healy et al. [8] found in Australian adults without diagnosed type 1 or type 2 diabetes that independent of total sedentary time and participation in moderate-to-vigorous intensity activity (MVPA), increased breaks in sedentary time were beneficially associated with waist circumference (WC), BMI, triglycerides, and 2-h plasma glucose. Thus, it may be prolonged, not interrupted sedentariness that may be most detrimental.

Until quite recently, metabolic risk factors were almost only observed in adulthood, but in recent years they are more frequently reported in children as well [9]–[11]. This is concerning as cardiometabolic risk factors track from childhood to adulthood [12]. In addition, cardiometabolic risk factors during adolescence predict the development of sub-clinical cardiovascular disease [13], coronary heart disease [14], and mortality in adulthood [15]. For effective prevention of type 2 diabetes and cardiovascular disease, it is, therefore, of great importance to also study the possible detrimental effects of prolonged sitting on metabolic health in children.

A recently published systematic review of the prospective relationship between sedentary behavior and health outcomes in young people [16] concluded that currently there is insufficient evidence for a prospective relationship between sedentary time and metabolic health. All but three of studies included in this review assessed sedentary behavior by self-report or parent report – and sedentary time was mainly operationalized as television time. The development of accelerometry as an objective measure of physical activity and sitting time has opened new, more complete as well as more objective possibilities for studying the health effects of sedentary time. Moreover, children engage in much more sedentary behavior than watching TV - a recent report on the ENERGY-project indicates that children spent almost as much time on computer-activities [17].

Therefore, we aimed to examine the cross-sectional association of objectively assessed sedentary time as well as self-reported TV and PC time with metabolic risk factors in a population-based sample of 10- to 13-year-old Dutch and Hungarian children. Our hypothesis was that there is an adverse relationship between both objectively assessed and self-reported sedentary time and metabolic risk in children aged 7–14 years, independent of their participation in moderate to vigorous intensity physical activity and adiposity.

## Methods

### Ethics Statement

Both the Medical Ethics Committee of the VU University Medical Center Amsterdam and the Scientific and Ethics Committee of Health Sciences Council in Hungary approved the study protocol. Each research team complied with the ethical procedure of their country. Both parents provided written informed consent and all children gave verbal consent.

### Study design and sample

Data were obtained as part of the ENERGY - EuropeaN Energy balance Research to prevent excessive weight Gain among Youth - project (www.projectenergy.eu) [18], [19]. The sample for the current analyses consists of girls and boys aged 10–13 years from two of the participating countries – Hungary and The Netherlands – where accelerometer data and blood samples were collected. Per country, three cities were selected with a different degree of urbanization (low, middle, and high tertile). Schools were randomly selected in the three cities to reach a representative sample of 1000 children per country, aged between 10 and 13 years old. The data collection took place between March and July 2010. Accelerometer data were collected from approximately 200 children per country. All children who participated in the accelerometer study were also asked to provide blood samples. The study design, selection criteria, and sample size are described in detail elsewhere [19].

### Procedure

Participants wore an ActiGraph accelerometer (models GT1M and Actitrainer) for at least six consecutive days. A 15 second epoch was used to capture the rapid transitions in activities typical for children [20]. Each child was asked to wear the ActiGraph at all waking times and remove the device only for water-based activities. A daily log sheet was provided to record any times the monitor was taken off and the reason for doing so. The raw data were analyzed using customized software (MeterPlus version 4.2 software from Santech, Inc. [www.meterplussoftware.com]). For inclusion in data analysis, each participant needed a minimum of ten hours per day of wearing time for at least three weekdays and a minimum of eight hours per day for at least one weekend day due to different sleep patterns at weekends [21]. We used the following exclusion criteria of non-wear time: 20 consecutive minutes of zero counts [22]. Wearing time was calculated by subtracting non-wearing time from 24 hours. A detailed description of the accelerometer protocol and data processing is described elsewhere [23].

We selected a cut-point of 100 counts per minute (cpm) as a cut-point for sedentary behavior since previous studies showed that this cut-point is the most appropriate one for quantifying time children spent on sedentary behavior [24]–[26]. We selected the cut-points from Treuth et al. [25] for moderate to vigorous activity intensity as well (MVPA≥3000 cpm).

TV time and PC time were assessed by self-report for weekdays and weekend days separately. Responses to each were summed to compute min/day of TV time and min/day PC time, respectively. Test-retest reliability over a one-week period (ICC>67) and relative validity compared with a cognitive interview indicated good construct validity of these items ICC>0.56 [30].

We collected data on body height and weight, and WC according to standardized procedures [19]. The children were measured in light clothing without shoes. Body height was measured with a Seca Leicester Portable stadiometer (accuracy of 0.1 cm). Weight was measured with a calibrated electronic scale SECA 861 (accuracy of 0.1 kg). WC was measured, as an indicator of abdominal fatness, four cm above the umbilicus [27] and recorded to the nearest 0.1 cm with the SECA 201 measuring band. Two readings of each measurement were obtained. If the two readings differed more than 1%, a third measurement was taken. All two or three measurements were recorded and the outlier was excluded during the data cleaning process and the mean of the two remaining recordings was calculated. Body mass index (BMI) was calculated for each child, and weight status (normal weight, overweight, obesity) was based on the International Obesity Task Force criteria [28].

We collected capillary blood samples in a validated collection kit developed for ambulatory purposes (Demecal, The Netherlands [29]).

The children were asked to fast from the evening before the morning of blood sampling. The fasting samples were taken between 8:00 and 8:30 A.M. and the children were offered breakfast afterwards. Fasting plasma glucose, C-peptide, total cholesterol, low density lipoprotein cholesterol (LDLC), high density lipoprotein cholesterol (HDLC), and triglycerides were determined.

### Analyses

All analyses were done with SPSS version 18.0. A relative metabolic risk score was computed from the following variables: WC, glucose, C-Peptide, HDLC, LDLC, and triglycerides. Each of these variables was standardized as follows: standardized value = ((value – group mean)/SD) stratified by gender [31]. The HDLC and C-peptide standardized value were multiplied by −1 to confer higher risk with increasing values for the purpose of calculating the metabolic risk. The metabolic risk score was calculated as the mean of the six standardized scores. The purpose of using a continuously distributed variable is to maximize statistical power. Gender-specific quartiles of sedentary time were calculated.

Data are presented as means ±SD. All variables were checked for normality. Gender differences in metabolic risk factors, sedentary time, and PA levels were assessed by t-tests (Table 1). Differences in metabolic indicators between the sedentary quartiles were checked by ANOVA. Differences between the 1^st^ and 4^th^ quartile of sedentary time, TV time and PC time, respectively, were examined by independent t-tests (Table 2). Significance levels for t-tests and ANOVA were set at p≤.05. The relationship between sedentary time and metabolic risk scores was examined using linear regression analysis adjusting for gender, country, number of sedentary bouts (when objectively assessed sedentary time was the outcome), MVPA, and WC (except with metabolic risk score as outcome)(Table 3).

**Table 1:** Baseline characteristics (means ± standard deviation) of 142 children (69 boys and 73 girls).

	Total N = 142	Girls N = 73	Boys N = 69
Age (yr)	12.2±0.6	12.2±0.6	12.2±0.7
Height (m)	155.9±7.6	155.7±6.6	156.2±8.5
Weight (kg)	48.5±10.8	47.0±9.3	50.1±12.1
BMI (kg/m^2^)	19.8±3.5	19.3±3.1	20.4±3.8
% Overweight (n)*	28 (39)	18 (13)	38 (26)
WC (cm)*	68.3±9.0	66.6±8.1	70.1±9.6
Total PA (Counts/15 sec)*	135±45	118±33	153±49
Light activity (min/day)*	264±56	254±54	275±57
MVPA (min/day)*	35±16	29±13	41±17
Sedentary time (min/day)*	485±66	502±59	468±70
# of sedentary bouts	69±22	68±21	70±23
Self-reported TV time (min/day)	116±64	114±64	118±65
Self-reported PC time (min/day)*	85±57	73±56	97±54
Glucose (mmol/l)	4.6±0.5	4.6±0.5	4.6±0.5
LDLC (mmol/l)	1.9±0.5	1.9±0.5	1.8±0.6
HDLC (mmol/l)	1.2±0.3	1.2±0.3	1.2±0.3
Tiglycerides (mmol/l)	0.8±0.5	0.8±0.3	0.8±0.6
C-peptide (nmol/l)	0.7±0.4	0.7±0.4	0.6±0.4
Metabolic risk score	−0.01±0.47	−0.01±0.44	−0.00±0.49

BMI = Body Mass Index, WC = waist circumference, MVPA = Moderate and Vigorous physical Activity, LDLC = low-density lipoprotein cholesterol, HDLC = high-density lipoprotein cholesterol.

* p≤0.05 for difference between boys and girls.

**Table 2:** Mean (± standard deviation) of metabolic indicators within quartiles of sedentary time in Dutch and Hungarian children.

	Sedentary time quartiles (mean±sd min/day)			
Objectively assessed sedentary time*	1^st^ 405±33	2^nd^ 457±29	3^rd^ 513±16	4^th^ 566±33
Self-reported sedentary time				
• TV time*	37±27	92±14	132±18	203±21
• PC time*	26±15	60±15	94±22	163±42
BMI (kg/m^2^)				
Objectively assessed sedentary time*	19±3	20±4	20±4	21±9
Self-reported sedentary time				
• TV time*	19±3	19±3	21±4	20±4
• PC time	19±3	19±3	21±4	20±4
WC (cm)				
Objectively assessed sedentary time*	66±8	68±10	69±10	70±8
Self-reported sedentary time				
• TV time*	66±7	68±10	69±10	70±9
• PC time	66±9	66±7	71±11	69±9
Glucose (mmol/l)				
Objectively assessed sedentary time	4.5±0.4	4.6±0.4	4.6±0.5	4.6±0.6
Self-reported sedentary time				
• TV time	4.4±0.4	4.7±0.5	4.7±0.4	4.6±0.4
• PC time	4.5±0.4	4.6±0.5	4.7±0.5	4.5±0.5
LDLC (mmol/l)				
Objectively assessed sedentary time	1.9±0.5	1.9±0.5	1.9±0.6	1.9±0.5
Self-reported sedentary time				
• TV time	1.9±0.5	2.0±0.5	1.9±0.6	1.8±0.5
• PC time	2.1±0.5	1.7±0.5	1.8±0.5	1.9±0.6
HDLC (mmol/l)				
Objectively assessed sedentary time	1.3±0.3	1.2±0.3	1.3±0.3	1.3±0.3
Self-reported sedentary time				
• TV time	1.2±0.3	1.3±0.4	1.3±0.3	1.2±0.3
• PC time	1.3±0.3	1.3±0.3	1.2±0.3	1.2±0.2
Triglycerides (mmol/l)				
Objectively assessed sedentary time	0.8±0.4	0.8±0.3	0.8±0.6	0.9±0.6
Self-reported sedentary time				
• TV time	0.7±0.4	1.0±0.4	0.8±0.5	0.8±0.4
• PC time	0.8±0.5	0.7±0.5	0.9±0.5	0.7±0.4
C-peptide (nmol/l)				
Objectively assessed sedentary time*	0.6±0.2	0.6±0.2	0.7±0.5	0.8±0.4
Self-reported sedentary time				
• TV time	0.6±0.3	0.8±0.5	0.7±0.3	0.6±0.2
• PC time	0.6±0.3	0.7±0.3	0.7±0.4	0.7±0.4
Metabolic risk score				
Objectively assessed sedentary time***	−.11±0.44	0.03±0.40	−0.03±0.48	.09±0.52
Self-reported sedentary time				
• TV time	−.12±0.42	.10±0.52	.02±0.43	.04±0.55
• PC time	−.01±0.47	−.16±0.46	.09±0.43	.02±0.47

BMI = Body Mass Index, WC = waist circumference, LDLC = low-density lipoprotein cholesterol, HDLC = high-density lipoprotein cholesterol.

* P<0.05 for difference between 1^st^ and 4^th^ quartile.

** p = 0.06 for difference between 1^st^ and 4^th^ quartile.

*** p = 0.09 for difference between 1^st^ and 4^th^ quartile.

**Table 3:** Regression (b-) coefficients and 95% Confidence Intervals (CI) as indicators of associations of sedentary time with individual metabolic risk factors and the metabolic risk score in Dutch and Hungarian children.

	Sedentary time	Self-reported TV time	Self-reported PC time
	(b-Coefficients [95% CI])	(b-Coefficients [95% CI])	(b-Coefficients [95% CI])
WC (cm)	0.003 (−0.03;0.04)	0.02 (−0.006;0.04)	0.02 (−0.005;0.05)
Glucose (mmol/l)	0.001 (−0.005;0.004)	0.002 (−0.001;0.005)	−0.001 (−0.003;0.004)
LDLC (mmol/l)	0.001 (−0.004;0.005)	−0.001 (−0.004;0.002)	−0.002 (−0.005;0.001)
HDLC (mmol/l)^a^	0.001 (−0.003;0.005)	−0.001 (−0.004;0.002)	−0.003 (−0.006;0.001)
Triglycerides (mmol/l)	0.000 (−0.004;0.005)	−0.001 (−0.004;0.002)	−0.00 (−0.003;0.003)
C-peptide (nmol/l)^a^	−0.003 (−0.006;0.001)	0.002 (0.000;0.004)	0.001 (−0.002;0.004)
Metabolic risk score	0.00 (−0.002;0.002)	0.001 (−0.001;0.002)	−0.00 (−0.001;0.002)

a recoded with higher values indicating higher risk.

WC = waist circumference, LDLC = low-density lipoprotein cholesterol, HDLC = high-density lipoprotein cholesterol.

Data are unstandardized regression coefficients (95% CI) and outcomes are expressed as standardized z-scores. All outcomes are adjusted for gender, country, number of sedentary bouts (when objectively assessed sedentary time was the outcome), Moderate and Vigorous Physical Activity, and waist circumference (except with metabolic risk score as outcome).

## Results

We collected blood from 210 children. We excluded blood data from 14 children because they were not in fasting state (n = 6) or the dilution factor was too low to report results (n = 8). Complete data of both accelerometry and blood values were available for 142 children (73 girls and 69 boys, 111 from Hungary and 31 from the Netherlands).

Table 1 shows the descriptive characteristics of the study sample. Children were on average 12 years old and 28% were defined as overweight (including obese) with a significant higher prevalence in boys than girls (38% versus 18%). Boys were significantly more physically active, reported higher PC time while their objectively assessed sedentary time was significantly lower. Participants spent on average 7.6 hours per day in sedentary behavior, 116±64 min/day on TV viewing and 85±57 min/day using the computer. Mean values of glucose, C-Peptide, HDLC, LDLC, and triglycerides were not significantly different between boys and girls.

Table 2 shows the metabolic indicators across gender-specific quartiles of objectively assessed sedentary time and self-reported TV and PC time. The metabolic risk score was lowest in the least sedentary quartile and highest in the most sedentary quartile. Glucose levels were lowest in the lowest quartile of TV viewing while LDL levels were highest in the lowest quartile for PC time. Comparing the 1^st^ and 4^th^ quartile of objectively assessed sedentary time, BMI, WC and C-Peptide levels were significantly higher in the most sedentary quartile, while the difference in metabolic risk score was borderline significant (p = 0.09). Comparing the 1^st^ and 4^th^ quartile of TV time, BMI was significantly higher in the most sedentary quartile, while the difference in WC score was borderline significant (p = 0.06).

Table 3 shows the associations of sedentary time with individual metabolic risk factors, and the metabolic risk score. All models were adjusted for gender and country. We found no significant association of sedentary time with metabolic risk.

## Discussion

Despite the fact that sedentary behavior is often suggested as an important risk factor for metabolic health independent of physical activity, there is very little research based on objective measures of both behavior and risk. The present study examined the relationship between objectively assessed sedentary time using accelerometers as well as self-rated TV time and PC time, and a range of metabolic indicators in healthy 10–12 year old Dutch and Hungarian children independent of MVPA and adiposity. Children in the most sedentary quartile of objectively assessed sedentary time had a significantly higher BMI, WC and C-Peptide levels (p<0.05) as well as an increased metabolic risk score (p = 0.09) compared to the least sedentary quartile. However, after adjustments for gender, country, number of sedentary bouts, and MVPA objectively assessed sedentary time or self-reported TV or PC time were not significantly associated with metabolic indicators. Also the pattern of how sedentary time was accumulated (number of sedentary bouts) was not related to metabolic indicators.

Only few previous studies examined the relationship between objectively assessed sedentary time and metabolic indicators. Sardinha et al. [2] studied 9- to 10-year old healthy, Portuguese children. However, they only examined insulin resistance - the homeostasis model assessment score - and found a positive association with sedentary time independent of MVPA. Participants in the Portuguese study were younger (9.8 versus 12.2 years) spent less time sedentary (315 versus 485 min/wk) and much more time in MVPA (177 versus 35 min/wk) compared to the Dutch and Hungarian children in the current study. Moreover, the Portuguese study used a longer epoch length (1 minute versus 15 seconds), a higher cut-point for sedentary behavior (500 versus 100 cpm) and a lower cut-point for MVPA (2000 versus 3000 cpm). This prohibits the comparability between studies. Similar to our findings, Carson and Janssen [32] found no association with overall volume or patterns of sedentary time with cardiometabolic health indicators in a large sample of 10–16 yr old US children using a similar cut point for sedentary behavior (<100 counts per minute). Conversely, they did find an independent association between self-reported TV time and metabolic risk factors, which is in contrast with our findings. A possible explanation for the contrasting findings is the larger age range (10–16 yrs) and the different metabolic indicators due to the unavailability of fasting blood samples in the study of Carson and Janssen.

Notable is the relatively high prevalence of overweight in boys (38%). We do not believe this has influenced our findings since BMI and overweight were no effect modifiers of the association between sedentary time and metabolic risk.

The following study strengths and limitations should be considered. Strengths include the objective and standardized measurement of sedentary time and metabolic indicators. Accelerometers are regarded as the gold standard for objective measurements of physical activity and sedentary time. However, even more accurate measures could have been obtained when combined with the use of inclinometers. Inclinometers such as the ActivPal (http://www.paltech.plus.com/products.htm) can also distinguish different postures and thus between lying, sitting and standing. Another strength is the focus on the accumulation of sedentary time adjusting for the number of sedentary bouts. Healy et al. [8] found in adults that it may be prolonged sitting, not interrupted sedentariness, that may be most detrimental. We could not confirm this finding in children. Since the current study and the one by Carson and Janssen [32] are the only studies examining the pattern of sedentary time, more research on this topic is needed.

Limitations include the cross-sectional design, thus limiting inferences of causality and its direction, and the lack of consensus on the cut-points used for defining sedentary time and MVPA. However, the cut-points we used have been validated in previous studies and the cut-point of 100 cpm for sedentary behavior has been shown to be most appropriate [24]–[26]. In addition, our sample size was relatively small since only about half of the parents provided informed consent for the blood collection. However, the non-responders to blood collection were not different to the current sample regarding, gender or BMI therefore we do not believe this will have lead to selection bias. With the current sample size of 142 subjects we were able to detect a standardized difference in glucose, HDLC, LDLC, triglycerides and C-Peptide levels of 0.25 with 0.8 power using a significance level of 0.05 with a two-tailed test. Finally, the results may not be generalizable to other populations of different age and other potential confounders not accounted for in the analyses, such as genotype and dietary habits, are not included but may bias the observed associations.

Our study – as most previous studies on this topic - included fasting blood samples. Nowadays children are seldom in fasting state. Thus, whether sedentary time impairs the metabolic response to food in children is another important question. Therefore, we recommend that future studies examine the association between sedentary time and postprandial values of glucose and lipids.

In conclusion, we found no evidence for a significant independent relationship between objectively assessed or self-rated sedentary time and metabolic indicators. Due to the scarcity of studies, we recommend further high quality prospective research using accurate measurement of both sedentary behavior as well as metabolic indicators to further investigate this relationship in young people.
